# Effects of Saffron Extract Supplementation on Mood, Well-Being, and Response to a Psychosocial Stressor in Healthy Adults: A Randomized, Double-Blind, Parallel Group, Clinical Trial

**DOI:** 10.3389/fnut.2020.606124

**Published:** 2021-02-01

**Authors:** Philippa A. Jackson, Joanne Forster, Julie Khan, Camille Pouchieu, Séverine Dubreuil, David Gaudout, Benjamin Moras, Line Pourtau, Florent Joffre, Carole Vaysse, Karène Bertrand, Hélène Abrous, David Vauzour, Julie Brossaud, Jean Benoit Corcuff, Lucile Capuron, David O. Kennedy

**Affiliations:** ^1^Brain Performance and Nutrition Research Centre, Northumbria University, Newcastle upon Tyne, United Kingdom; ^2^Activ'Inside, Beychac et Caillau, France; ^3^ITERG, Nutrition-Health & Lipid Biochemistry Department, Canéjan, France; ^4^Faculty of Medicine and Health Sciences, Biomedical Research Centre, Norwich Medical School, University of East Anglia, Norwich, United Kingdom; ^5^Hormone Laboratory, Nuclear Medicine, CHU Bordeaux, UMR INRA 1286, University Bordeaux, Bordeaux, France; ^6^Univ. Bordeaux, INRAE, Bordeaux INP, NutriNeuro, UMR 1286, Bordeaux, France

**Keywords:** saffron, depression, anxiety, heart rate variability, crocetin

## Abstract

Anxiety, stress, and low mood are closely related and may contribute to depressive symptoms. Among non-pharmacological solutions to improve subclinical mood symptoms and resilience to stress, natural products such as saffron—identified as promising following preliminary beneficial effects in major depressive disorder—represent a relevant strategy. This study aimed to assess the efficacy of 8 weeks' supplementation with 30 mg standardized saffron extract on emotional well-being in healthy adults with subclinical feelings of low mood and anxiety and/or stress and evaluate the acute effect of saffron in response to a lab-based psychosocial stressor. The study adopted a double-blind, randomized, parallel groups design in which 56 healthy male and female individuals (18–54 years) received either a saffron extract or a placebo for 8 weeks. Chronic effects of saffron on subjective anxiety, stress, and depressive feelings were assessed using a questionnaire battery [including Profile of Mood State-2, (POMS)] and acute effects in response to a lab-based psychosocial stressor were measured through psychological and physiological parameters. Urinary crocetin levels were quantified. Participants who received the saffron extract reported reduced depression scores and improved social relationships at the end of the study. Urinary crocetin levels increased significantly with saffron supplementation and were correlated with change in depression scores. The typical stress-induced decrease in heart rate variability (HRV) during exposure to the stressor was attenuated following acute saffron intake. Saffron extract appears to improve subclinical depressive symptoms in healthy individuals and may contribute to increased resilience against the development of stress-related psychiatric disorders.

**Clinical trials number:** NCT03639831.

## Introduction

Depression is one of the most prevalent psychiatric disorder and has been estimated to affect over 300 million people worldwide, representing about 4.4% of the world's total population (1). Actual figures are likely to be higher as this rate only includes diagnosed cases with subclinical levels of mood disorders being also highly prevalent (2). Pharmacological treatments exist, but a number of articles have emphasized the inability of antidepressant medication to consistently demonstrate superiority to placebo, in patients with mild or moderate symptoms (3). Side effects of such treatments, including nausea and drowsiness, are also common, especially at medium to high doses in the case of selective serotonin reuptake inhibitors (SSRIs) (4). Alternative treatments, such as natural products or exercise for combating mood disorders, are therefore being explored (5).

Among natural products, saffron, produced from the dried stigma of *Crocus sativus* L., a perennial herb member of the *Iridaceae* (Iris) family, appears a promising candidate. Saffron stigmas are naturally rich in four major bioactive compounds: crocin, crocetin (the hydrolysis product of crocin), picrocrocin, and safranal (6) and are traditionally used in Asian (particularly Persian) medicine to treat a range of physical ailments including menstrual disorder, inflammation, and depressive symptoms (7, 8). A meta-analysis of five clinical trials revealed a treatment effect of saffron compared with placebo and similar antidepressant efficacy when compared with antidepressant medications (fluoxetine, imipramine), at a dose of 30 mg/day (all studies) for at least 6 weeks in participants with major depressive disorder (9). The extent to which the antidepressant effects of saffron are replicated in individuals experiencing low mood is currently unknown.

Certain environmental factors may be involved in the onset and development of depressive symptoms. Among those factors, chronic stressful life events during adulthood may activate or amplify the expression of depressive symptoms (10). While some people exposed to stressful events do not show signs or symptoms of depression, others exposed to psychological stress are vulnerable to depression (11). In response to psychological stress, a series of neurobiological changes occur; abnormalities in which are commonly reported in depressive patients (12). Interestingly, saffron may reduce plasma levels of corticosterone in response to stress in both acute and chronic rodent studies (13, 14), suggesting potential modulation of the hypothalamic–pituitary–adrenal (HPA) axis following this extract. In humans, to our knowledge, no study has evaluated the effect of saffron in response to induced acute stress. A single study has shown that healthy female humans exposed to saffron aroma for 20 min also experienced a decrease in salivary cortisol levels, which was accompanied with a decrease in anxiety measured using the State–Trait Anxiety Inventory (STAI) (15).

Overall, the evidence suggests that the efficacy of saffron with regards to the modulation of mood may extend beyond clinical populations and might also include the attenuation of stress responses. This research is important given that the incidence of major depression has been shown to be higher in patients with diagnosed subclinical depression (16). Therefore, the aim of the present study was (1) to assess the chronic effect of saffron on emotional well-being evaluated by a series of questionnaires in healthy adults self-reporting low mood and (2) to evaluate the acute effect of saffron extract in response to a psychosocial stressor through psychological and physiological parameters.

## Materials and Methods

### Design

This study adopted a randomized, placebo-controlled, double-blind, parallel groups design, in which the acute and chronic effects of saffron extract and placebo were assessed before, during, and after a laboratory stressor, the Observed Multitasking Stressor (OMS) on days 1, 14, 28, and 56 after treatment consumption. This study was conducted according to the guidelines laid down in the Declaration of Helsinki, and all procedures involving human participants were approved by the Northumbria University's Department of Psychology Ethics Committee (Ref: 1028). Written informed consent was obtained from all participants. The trial is registered at ClinicalTrials.gov (NCT03639831).

### Participants

Seventy-three participants aged 18–60 years who self-reported feelings of anxiety and/or stress and low mood in their daily lives were recruited within Newcastle-upon-Tyne and the surrounding areas. The study was advertised using emails distributed to the research center's participant database and Northumbria University's internal distribution lists, along with adverts placed on social media. Participants who complied with the diagnosis criteria (using self-report questionnaires) of any psychological pathology (e.g., depression, generalized anxiety disorder) or diagnosed (by a medical practitioner) for such pathology within the previous 3 years were not eligible. As well, exclusion criteria were the following: body mass index (BMI) ≤18.5 or ≥30 kg/m^2^; uncorrected visual impairment; food allergies/insensitivities; an hormonal status likely to induce an unstable/fluctuating emotional state (e.g., menopausal transition); presence of life event likely to induce unstable/fluctuating emotional state (e.g., change of professional function/ situation, death of a family member, divorce, surgery); high blood pressure (systolic over 159 mm Hg or diastolic over 99 mm Hg); pregnant women, seeking to become pregnant, or lactating; worked night shifts; engaged in high levels of physical activity (to avoid bias in the salivary cortisol measures); consumed >500 mg caffeine per day; current smokers; and dietary supplement use 2 weeks before enrolment. A number of health conditions were also excluded; a full list of inclusion/exclusion criteria is presented in the Online Supplementary Material.

**Figure 1:**
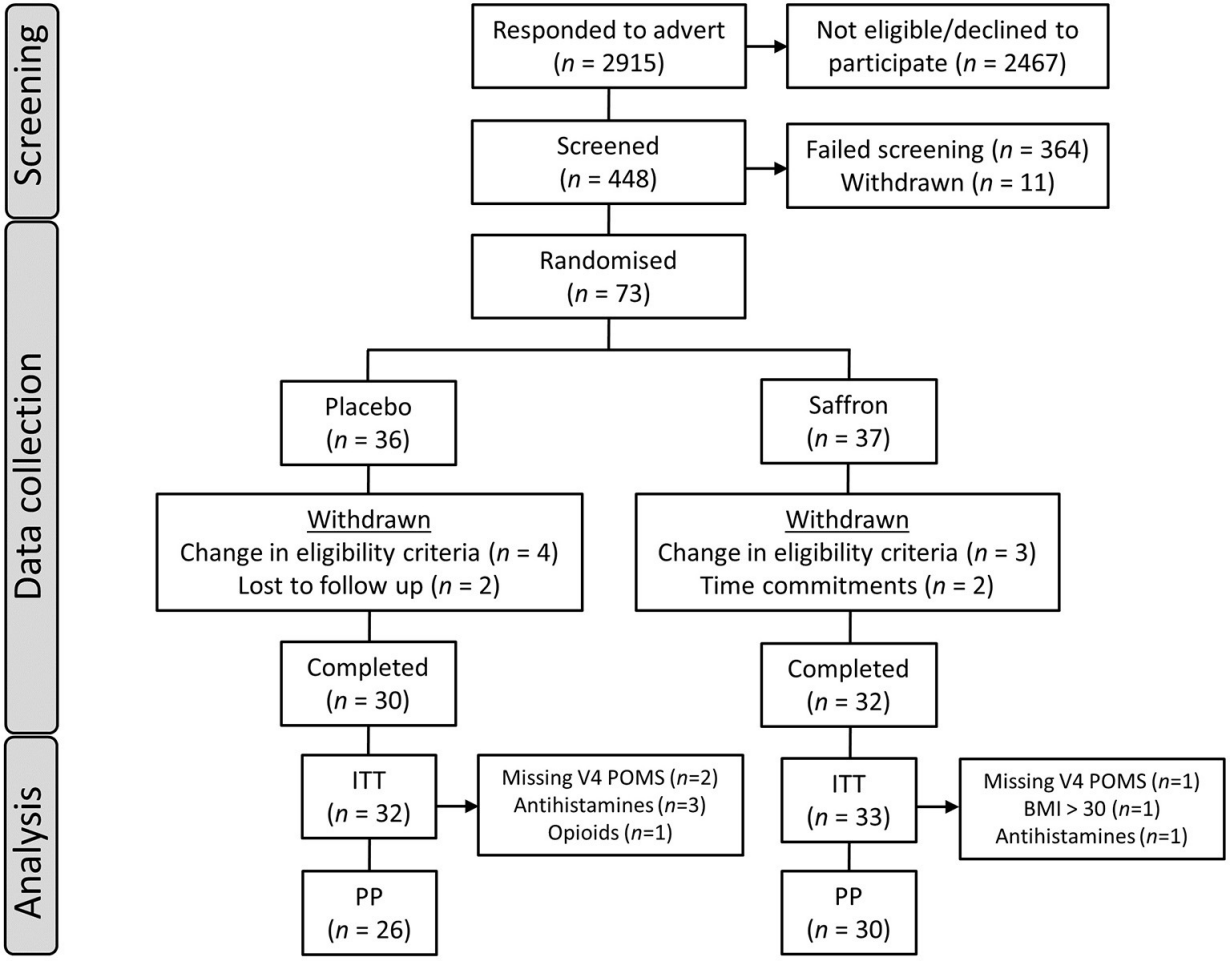
Participant disposition flowchart. ITT, intent to treat population; PP, per protocol population; POMS, Profile of Mood States questionnaire; BMI, body mass index.

In order to recruit a sample of participants who may be experiencing subclinical levels of depressive symptoms, participants were administered a number of questionnaires at the screening visit with the following exclusion thresholds in a first attempt: >36 on the Penn State Worry Questionnaire (PSWQ) (17); >13 on the Perceived Stress Scale (PSS-10) (18); <10 on the Generalized Anxiety Disorder 7-item questionnaire (GAD-7) (19); <10 on the Patient Health Questionnaire 9-item (PHQ-9) (20), and ≥35 on the State-Trait Anxiety Inventory State (STAI) (21). However, due to initial difficulties with recruitment, the following inclusion criteria were subsequently adopted: total score ≥40 on the Profile of Mood States 2 (POMS) (22), <16 on the GAD-7 questionnaire, and ≤10 on the PHQ-9 questionnaire. Seven participants who were randomized to treatment according to the initial exclusion criteria were later withdrawn from the study. Fifty-six participants were included in the final per-protocol analysis set (see Figure 1). All participants gave their written informed consent before inclusion in the study and were compensated £230 for their time and any travel costs upon completion of the study. Data were collected between November 2017 and November 2018.

Participant demographics and baseline characteristics are shown in Table 1.

**Table 1:** Baseline characteristics by treatment group.

**Measure**	**Treatment**	**Mean**	**SD**	***p***
Sex ratio (male/female)	Placebo	6/20		
	Saffron	10/20		
Age (years)	Placebo	28.62	9.49	0.80
	Saffron	28.00	9.11	
Education (years)	Placebo	16.69	2.51	0.72
	Saffron	16.98	3.39	
Cigarettes (number/week)	Placebo	0.19	0.98	0.32
	Saffron	0.90	3.45	
Fruit and veg consumption (portions/day)	Placebo	4.21	1.52	0.35
	Saffron	3.82	1.62	
Alcohol consumption (units/day)	Placebo	0.72	0.80	0.70
	Saffron	0.81	0.69	
Caffeine consumption (mg/day)	Placebo	176.08	87.44	0.48
	Saffron	198.03	133.79	
Systolic BP (mm/Hg)	Placebo	118.56	11.55	0.57
	Saffron	116.67	13.00	
Diastolic BP (mm/Hg)	Placebo	77.69	8.09	0.26
	Saffron	75.13	8.56	
Heart Rate (beats per minute)	Placebo	71.25	11.15	0.43
	Saffron	68.98	10.00	
BMI (kg/m^2^)	Placebo	23.15	2.68	0.81
	Saffron	22.97	2.91	
GAD-7 Score	Placebo	6.58	3.40	0.70
	Saffron	6.90	2.92	
PHQ-9 Score	Placebo	5.92	2.87	0.25
	Saffron	6.77	2.56	
POMS Score	Placebo	64.60	22.27	0.69
	Saffron	62.34	18.31	

*BP, blood pressure; GAD-7, Generalized Anxiety Disorder-7 item questionnaire; PHQ-9, Patient Health Questionnaire-9 item questionnaire; POMS, Profile of Mood States. p-values were derived from independent measures t-tests (or Mann–Whitney U-tests in the case of non-normally distributed data)*.

### Treatment and Dosing Schedule

Participants were randomly allocated to receive 30 mg saffron extract (Safr'Inside™) standardized in Safromotivines™ [a blend of more than 25 active compounds, including safranal >0.2% (analyzed with HPLC)] or a placebo. Each saffron capsule contained 15 mg saffron extract plus 345 mg maltodextrin, and each placebo capsule contained 350 mg maltrodextrin. Treatments were randomized in accordance with a computer-generated randomization list that was generated by an academic colleague at Northumbria University who had no further involvement in the study. Treatments were delivered to the investigational site identified only by their randomization code. Treatments were provided by Activ'Inside in bottles and were identical in appearance to ensure that participants remained blind to the treatment they had received. Non-stratified randomization of participants took place at the first testing study visit (day 1), where they were allocated to the next sequential randomization number. Blinding remained in place until all of the data, bar the urinary crocetin data, had been analyzed according to the statistical analysis plan.

Participants consumed the first dose (two hard shell capsules) of their 56-day treatment within the laboratory during their visit (day 1). They then continued to consume this treatment daily, at home. One capsule was taken in the morning and another in the evening, at least 30 min after a meal. Participants completed a dosing diary each day with the time of each capsule consumption noted. These instructions were emphasized at training, at the end of the acute lab visit days, and were printed on their treatment bottle label. Treatment on days 14 (±2 days), 28 (±3 days), and 56 (±3 days) was again consumed in the laboratory (two capsules) prior to completing the OMS.

### Observed Multitasking Stressor

The OMS incorporates two elements that have previously been shown to engender a stress response in laboratory studies: extended multitasking and social evaluation. The OMS has previously been shown to provoke a psychological stress response across repeated administrations (23). Briefly, the OMS comprised verbal completion of three serial subtraction tasks (3, 7, and 17's) for 4 min each (12 min in total). Participants were instructed to count backwards from a given randomly generated number between 800 and 999 aloud, as quickly as possible. Performance of the task was scored for the total number of correct subtractions and incorrect subtractions. In the case of incorrect responses, subsequent responses were scored as correct if they were correct in relation to the new number. During the serial subtraction tasks, participants also completed a computerized tracking task, in which they were required to use the mouse to move a cursor to attempt to track an asterisk that followed a smooth, random on-screen path; participants were instructed to keep the cursor as close to the asterisk as possible.

**Figure 2:**
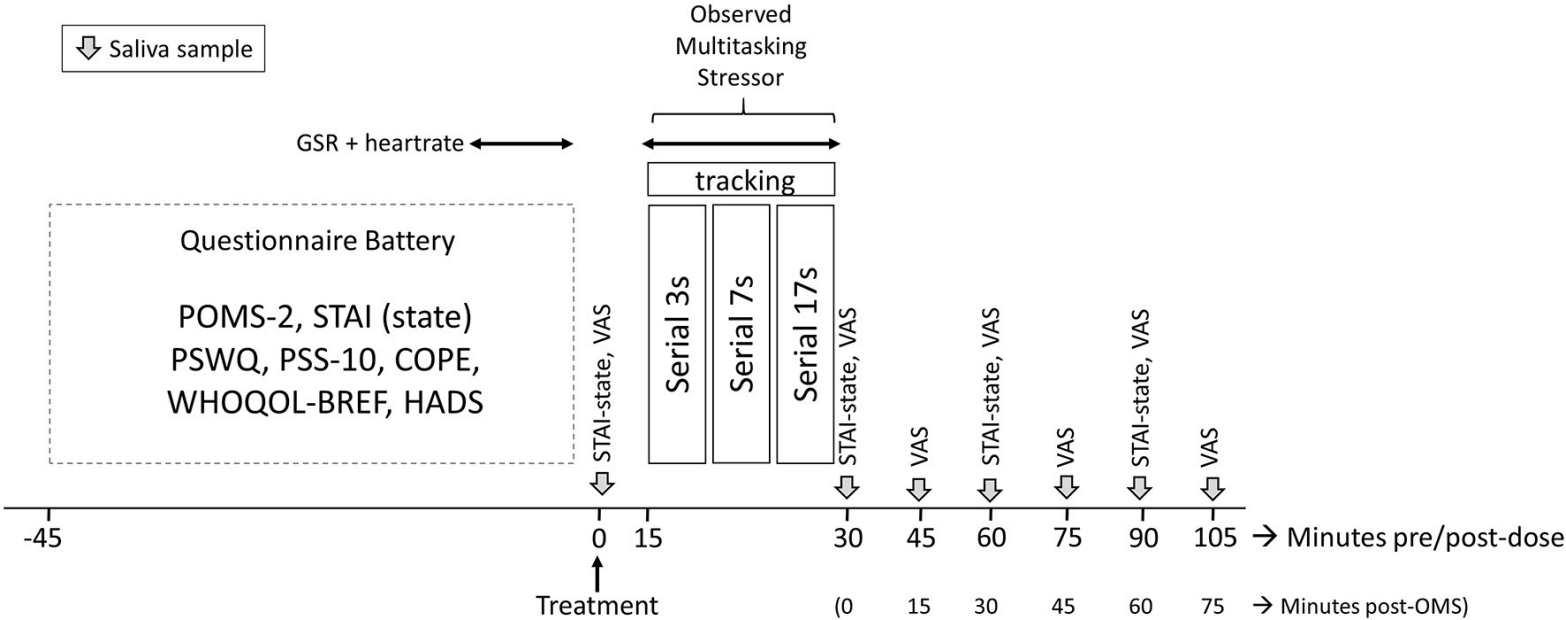
Testing timeline of the study visits. Participants completed a battery of questionnaires predose on arrival; the State–Trait Anxiety Inventory (STAI)-State, visual analog scales (VAS), and saliva samples were completed preobserved multitasking stressor (OMS). Galvanic skin response (GSR) and heart rate were collected for 5 min during completion of the questionnaire battery and again during the OMS. Collection of saliva samples and completion of VAS was repeated immediately and at 15-min intervals poststressor, respectively; the STAI-State was completed immediately and at 30-min intervals. POMS-2, Profile of Mood States 2; PSWQ, Penn State Worry Questionnaire; PSS-10, Perceived Stress Scale-10; WHOQOL-BREF, World Health Organization Quality of Life questionnaire; HADS, Hospital Anxiety and Depression Scale.

These tasks were performed in front of a panel of three “judges” who maintained a neutral demeanor throughout the assessment. The computer screen, showing the tracking task, was projected onto a screen to give the impression that the panel was closely monitoring progress. Before and immediately after completing the OMS, mood was assessed with the STAI (State) and computer-delivered visual analog scales (VAS) indicating the participants' current level of stress, anxiety, relaxation, and calmness (see below). These measures of mood were also repeated every 15 min (VAS) or 30 min (STAI, State) after completion of the stressor, as per Figure 2. A full description of the OMS can be found in the Online Supplementary Material.

### Mood and Well-Being Measures

Before undergoing the OMS, participants completed a number of psychological questionnaires: the POMS-2 (22), STAI (Trait) (21), the PSWQ (17), the PSS-10 (18), the COPE Inventory (COPE) (24), the Hospital Anxiety and Depression Scale (HADS) (25), and the World Health Organization Quality of Life questionnaire (WHOQOL-BREF) (26). The POMS “total mood disturbance” (TMD) score was the primary outcome measure.

The VAS that were completed before and after the OMS were presented using the Computerized Mental Performance Assessment System (COMPASS, Northumbria University, Newcastle upon Tyne, UK). Line scales anchored “not at all” (left-end) and “extremely” (right-end) were presented to participants who were required to mark a cross on a line using a mouse to indicate their current level of stress, anxiety, relaxation, and calmness. These were scored as percentage along the line from left to right.

### Biological Samples Analysis

#### Salivary Cortisol and Alpha Amylase Analysis

Saliva samples were obtained using salivettes (Sarstedt Ltd, UK) to measure cortisol and alpha-amylase response. Seven samples were collected per testing visit with a sample taken prior to completion of the OMS and another immediately after. Remaining samples were collected at 15-min intervals post stressor. Once collected, samples were spun down at 1,000 g for 2 min. Samples were transferred into Eppendorfs and frozen at −80°C until analysis. Before assaying, the samples were thawed; cortisol and α-amylase levels were measured using ELISA kits and carried out according to the instructions of the supplier (Salimetrics Ltd, UK). The coefficients of variability for the cortisol and α-amylase assays were 20 and 15%, respectively.

#### Urinary Crocetin Analysis

Samples were collected from the first urination in the morning prior to attending the lab for each visit. Participants were instructed to urinate into a measuring jug, note down the total amount in milliliters (ml), and the time of collection. If participant needed to urinate during the night, this was collected using the same method as above; however, it was then transferred into a storage vessel and refrigerated overnight. In the morning, it was mixed with the first urination of the morning, and the entire vessel was brought to the lab. Participants were provided with freeze-able pouches to keep their samples cool on their journey to the lab. Samples were frozen at −80°C and shipped to France for crocetin analysis. For identification and quantification of urinary crocetin, samples of 10 participants receiving placebo and 20 participants receiving saffron extract were saponified, extracted with ethyl acetate, and were analyzed by ultrahigh performance liquid chromatography tandem mass spectrometry (UHPLC-MS/MS) (ThermoFisher Scientific, Courtaboeuf-France) according the modified chromatographic conditions of Zhang et al. (27). Briefly, chromatographic separation of the analytes was performed on a C18 column (100 mm × 2.1 mm, 3 μm, Atlantis®T3, Waters). The aqueous mobile phase (solvent A) contained 0.1% formic acid, while the organic mobile phase (solvent B) was acetonitrile. The negative mode was selected, and the analytes were quantitatively monitored using multiple reaction monitoring (MRM). The MRM transition for crocetin was m/z 327.145 → 239.30. The ion spray voltage was optimized and maintained at 3,000 V. The crocetin concentration in urine samples was calculated using a standard linear curve at several crocetin concentration (from 0.09 to 0.90 ng/ml). The limit of detection and quantification were 0.04 and 0.13 ng/ml, respectively, with an intra-assay precision CV of 4% and an interassay variability and precision CV of 8%. The level of creatinine was determined by a coupled enzymatic and colorimetric essay (Sigma-Aldrich, St Louis, USA) for each urinary sample to take into account different urine concentrations between volunteers. The quantity of urinary crocetin was therefore expressed in nanograms of crocetin per milligram of creatinine.

### Galvanic Skin Response and Heart Rate

Galvanic skin response (GSR) and heart rate (HR) were measured using the Vilistus Digital Sampling Unit (Durham Systems Management Limited, UK). The GSR sensors, which measured relative changes in skin conductance, were attached to the middle and forth fingertips of the participants' non-dominant hand using Velcro straps. The HR sensor clip, which measured blood volume pulse, was placed on the tip of the index finger or thumb of the non-dominant hand. These sensors were attached at least 1 min prior to the commencement of recording to allow for stabilization of the readings. The unit measured at a rate of 32 or 128 samples per second for GSR and HR, respectively. The software that accompanies this device also calculates a number of HRV measures including root mean square of the successive differences (RMSSD) HRV index (an indication of the variability within the heart rate across the session calculated from the inter-beat interval plot), pnn50 (mean number of times per hour in which the change in consecutive normal sinus intervals exceeds 50 ms), and a stress index (the ratio between the parasympathetic and sympathetic tone). No sensitivity data were provided by the manufacturer however this device has been successfully used by our lab and others in the assessment of changes in electrodermal activity in response to an intervention (23, 28, 29).

### Satisfaction and Tolerance Questionnaire

At the final visit, participants were asked to complete an in-house product satisfaction and tolerance questionnaire. The questionnaire aimed to ascertain if participants had experienced any changes in their libido and any beneficial effects of taking the treatment on their mood and well-being. It also inquired whether the participants found the product to be well-tolerated and easy to use. Finally, participants were asked to guess which treatment they had been given.

### Procedure

Participants were required to attend the Brain, Performance and Nutrition Research Centre (Northumbria University) for five visits. The first visit comprised a screening and training session; once written informed consent had been obtained, participants were screened according to the inclusion/exclusion criteria. Eligible participants then provided lifestyle and demographic data, and their height, weight, and blood pressure were measured. They completed a short training session in which they practiced the serial subtraction tasks, the computerized tracking task, and computerized VAS, along with a practice saliva sample (not analyzed), and completed the STAI (Trait) questionnaire. At the end of this visit, participants were provided with urine collection containers and instructions for urine collection.

Within 2 weeks of the screening visit, participants returned to the laboratory for their first testing visit at an agreed time in the afternoon that remained consistent across all testing visits. Participants completed urine collection on the morning of the testing visit and brought this sample with them to the laboratory. Participants arrived at the laboratory having consumed their normal breakfast and lunch and having consumed their normal amounts of caffeinated products, which was documented on a food diary. The diary contained basic dietary information for the day of the testing visit as well as the previous day including type of food/drink consumed and when. These data were used to check caffeine consumption restrictions and that an equivalent diet was consumed prior to all visits. Once participants arrived at the lab, they were not permitted to eat any food or chewing gum. Continued compliance with the inclusion/exclusion criteria was assessed via the Case Report Form. Participants then completed the POMS, STAI (State), PSWQ, PSS-10, HADS, COPE, and WHOQOL-BREF.

Participants consumed their first dose of the allotted treatment and completed the pre-OMS STAI (State), along with the computerized VAS. Following this, they provided a saliva sample. At 15 min postdose, participants were taken to an “interview” room where they underwent the OMS for 15 min in front of a panel of three observers. GSR and HR were measured during the OMS following a predose baseline assessment of 5 min that was collected during completion of the questionnaires. Saliva samples were provided immediately after the OMS and at 15-min intervals up to 75 min post OMS (i.e., 105 min post dose) with the VAS being completed at the same time points. The STAI (State) was completed immediately after the OMS and at 30 and 60 min post OMS. The full assessment timeline during all study visits is shown in Figure 2.

In the interim periods between each of the testing visits, participants were instructed to take their daily treatment and complete a treatment diary on a daily basis. Participants were reminded to consume the same foods and beverages consumed on the day 1 testing visit for all subsequent testing visits. The procedure during the subsequent three visits was identical to that of day 1, with the exception that participants returned treatment diaries and unused capsules. On day 56, the STAI (Trait) was completed along with a tolerance and satisfaction questionnaire.

### Statistical Analysis

All outcomes were analyzed using SPSS (version 24.0, IBM corp.) unless otherwise specified.

#### Sample Size Calculation

To detect a difference between placebo and saffron of 22 points (SD = 30) on the Total Mood Disturbance outcome of the POMS (the primary endpoint) (22), with a two-sided 5% significance level and a power of 80%, a total sample size of 72 participants was deemed necessary. This total included allowance for an anticipated 15% dropout rate.

#### Treatment Compliance and Tolerance, Blinding, and Baseline Differences

Independent measures *t*-tests (or Mann–Whitney *U*-tests in the case of non-normally distributed data) were used to compare treatment groups at baseline and treatment compliance. A chi-squared test was used to assess the success of the double-blind procedure and to analyze the tolerance data.

#### Chronic Assessment of Mood and Well-Being Questionnaires

For the pre-OMS questionnaires (POMS, HADS, STAI-State, PSWQ-2, PSS-10, COPE, and WHOQOL-BREF), change from day 1 scores for days 14–56 were analyzed using the MIXED procedure in SPSS for between-group differences including the terms Treatment and Visit and their interaction as a fixed factors. With regard to the primary outcome measure, the between group difference at day 56 on the POMS-TMD score was analyzed using Bonferroni-adjusted pairwise comparisons following the linear mixed model analysis.

#### Acute Response to the OMS

For the STAI (State) and VAS data, change from baseline scores (post-OMS value minus pre-OMS value) were analyzed for between group differences using the MIXED procedure including the terms Treatment, Assessment, and Visit and their interactions as fixed factors.

Change from baseline values for GSR (μSiemens) and HR (beats per minute) (during OMS value minus pre-OMS value) were analyzed for between-group differences including the terms Treatment, Task, and Visit and their interactions as fixed factors. The number of total correct answers and errors for serial subtractions tasks and the tracking cost for tracking task were analyzed including the terms Treatment, Visit, and Task and their interactions as fixed factors. For the HRV outcomes, due to significant deviation from normality of the residuals, some HRV measures were log or square root transformed. Between-group differences in transformed or natural HRV measures during the OMS were analyzed using a linear mixed model including the terms Treatment and Visit, the interaction Visit × Treatment, and the baseline HRV measures as fixed factors.

#### Salivary Cortisol and α-Amylase

Incremental area under the curve (iAUC) and net iAUC were calculated for salivary cortisol and salivary alpha-amylase. First, the incremental AUC (iAUC), i.e., the area over the limit of quantification under the curve and ignored area beneath the curve, was calculated with the linear up-log down method of non-compartmental analysis, with the PKsolver add in for Excel (30). Second, the net iAUC, i.e., the positive area minus the negative area over the pre-OMS value, was calculated using the linear trapezoidal method of non-compartmental analysis (31). iAUC, net iAUC, and the maximum change from sample 1 were analyzed for between group differences including the terms Treatment and Visit and their interaction as fixed factors. Wilcoxon signed-rank test was used in *post-hoc* comparisons to assess the difference at each visit, within treatment group. Time to maximum concentration (Tmax) was analyzed non-parametrically using the Friedman test to assess equivalence of ranks within each treatment group across the four visits.

#### Urinary Crocetin

Urinary crocetin (ng/mg of creatinine) under the limit of detection (LOD) were imputed by LOD/$\sqrt{2}$ (32). Urinary crocetin was analyzed with a linear mixed model with Treatment, Visit, and their interaction, after log-transformation due to non-normally distributed data. Spearman correlation between variation in urinary crocetin (difference day 56–day 1) and variation in POMS depression T score (difference day 56–day 1) was performed.

#### OMS Validation

In order to confirm that participants found the OMS protocol stressful, STAI-State, VAS anxiety, salivary cortisol, and α-amylase were compared before, during, and after the OMS. The models were set up as above, including the terms Treatment, Visit, and Assessment and their interactions as fixed factors. Subject was included as a random factor.

## Results

### Treatment Compliance and Tolerance, Blinding, and Baseline Differences

There were no significant between-group differences on any baseline demographic measure (Table 1, all *p* > 0.05). Overall compliance, assessed by pill count, was excellent (97% in the placebo group and 96% in the saffron group); a *t*-test confirmed that compliance was not significantly different between the treatment groups (*p* = 0.60). Success of blinding was confirmed via treatment guess at the end of the study. Fifty-two percent of participants in the placebo group and 41% of participants in the saffron group believed they had received placebo. Chi-squared analysis confirmed this to be a non-significant difference (*p* = 0.40). A lack of any between-group differences on the satisfaction questionnaire indicated that participants who received saffron found the product to be as tolerable as the placebo, and a similar number of adverse events were reported by each group (none were classified as serious).

Day 1 between-group differences were analyzed for the pre-OMS questionnaire and GSR outcomes. Results indicated that participants who received saffron had significantly higher religious coping (*U* = 462.5, *p* = 0.005) and positive reinterpretation coping [*F*_(1,53)_ = 4.81, *p* = 0.03] on the COPE questionnaire before treatment.

### Effect of Chronic Saffron Supplementation on Emotional Well-Being

**Figure 3:**
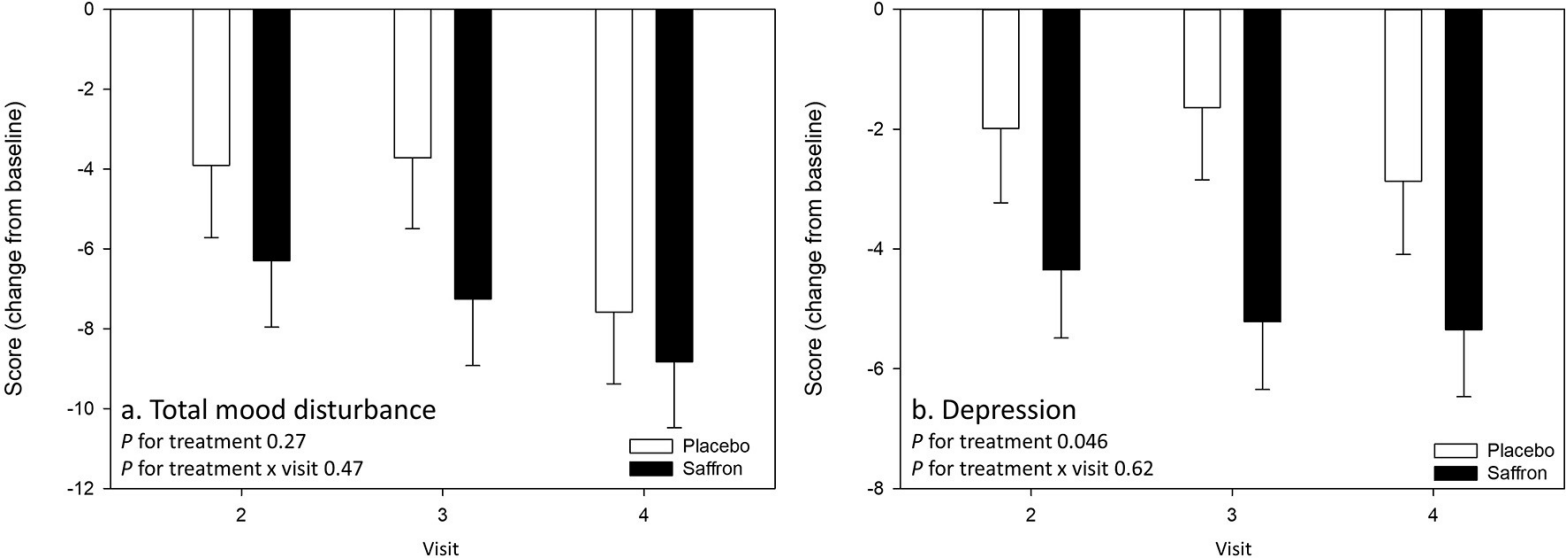
Change from baseline day 1 responses on the primary outcome measure **(A)** Profile of Mood States (POMS) Total mood disturbance and **(B)** the POMS Depression subscale by treatment and visit. No effects were observed on the primary outcome measure. A main effect of treatment was observed for depression (*p* = 0.05). Estimated means ± standard error are shown.

There was no effect of treatment nor treatment × visit interaction on the primary outcome measure, T-score of POMS Total mood disturbance. However, there was a significant effect of treatment on the POMS Depression subscale (T-score), with participants who received saffron seeing a greater reduction in their subjective experience of depressed mood from predose day 1 across the visits than the placebo group [*F*_(1,51.58)_ = 4.18, *p* = 0.05]. See Figure 3.

**Figure 4:**
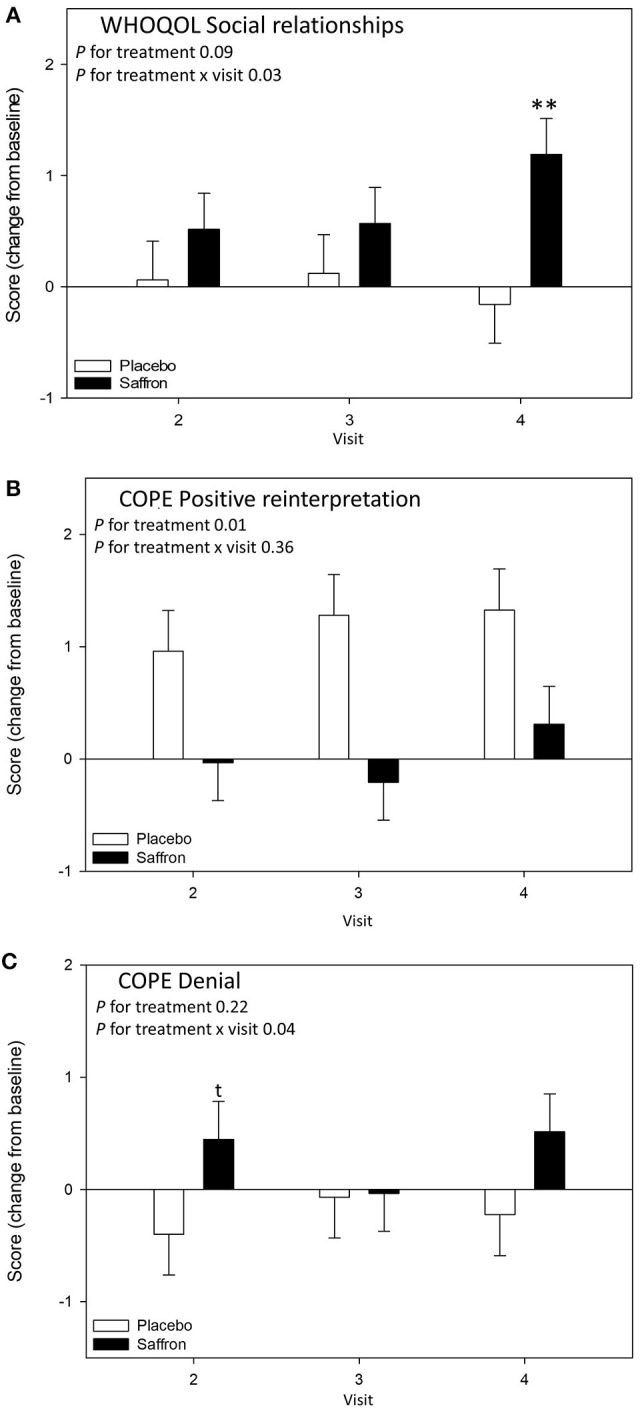
Change from baseline day 1 responses on the chronic mood/well-being questionnaires by treatment and visit; **(A)** World Health Organization Quality of Life Questionnaire (WHOQOL) Social relationships subscale, **(B)** COPE questionnaire Positive reinterpretation subscale, and **(C)** COPE questionnaire Denial subscale. Estimated means ± standard error are shown. **p < 0.01; *t, p* < 0.10.

A significant Treatment × Visit interaction was observed for the domain Social Relationships of the WHO quality of life questionnaire [*F*_(2,63.65)_ = 3.88, *p* = 0.03]. Pairwise comparisons revealed that participants receiving saffron had a higher social relationship score compared to placebo at 56 days post-supplementation (*p* = 0.007) (see Figure 4A).

A significant treatment effect was observed for positive reinterpretation coping [*F*_(1,51.71)_ = 7.25, *p* = 0.010] with participants who received saffron having an attenuated increase in positive reinterpretation compared to placebo (see Figure 4B). A significant Treatment × Visit interaction was observed for the denial coping strategy [*F*_(2,65.71)_ = 3.39, *p* = 0.04]. Pairwise comparisons revealed a borderline difference on day 14; participants who received saffron had a higher score compared to placebo at 14 days post-supplementation (*p* = 0.09) (see Figure 4C).

**Figure 5:**
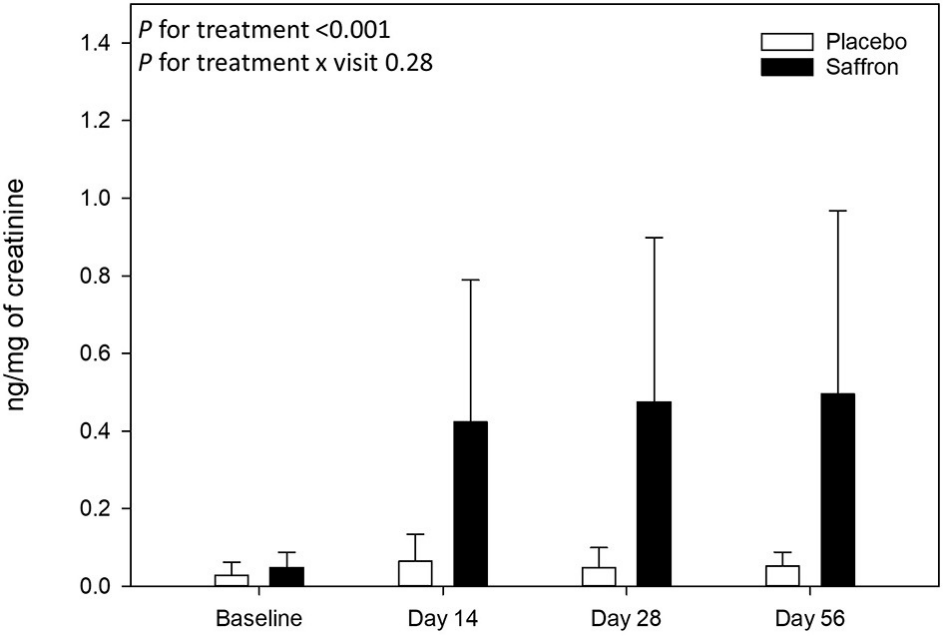
Urinary crocetin levels collected at baseline and at 14, 28, and 56 days post-supplementation. Data are means ± standard deviation of raw values. Urinary crocetin levels were expressed by milligrams of creatinine.

Finally, chronic saffron supplementation induced a significant change in urinary crocetin level (*p* < 0.0001) (see Figure 5). Indeed, participants who received saffron had a higher mean level of urinary crocetin (0.46 ± 0.42 ng/mg of creatinine) than those receiving placebo (0.05 ± 0.05 ng/mg of creatinine) after supplementation, all visits taken together (days 14–56). A significant correlation between variation in POMS depression T-score and variation in urinary crocetin level (day 56–day 1) was observed (Spearman ρ = −0.50, *p* = 0.008).

### Effect of Saffron in Response to a Psychosocial Stressor

#### Validation of the OMS

**Figure 6:**
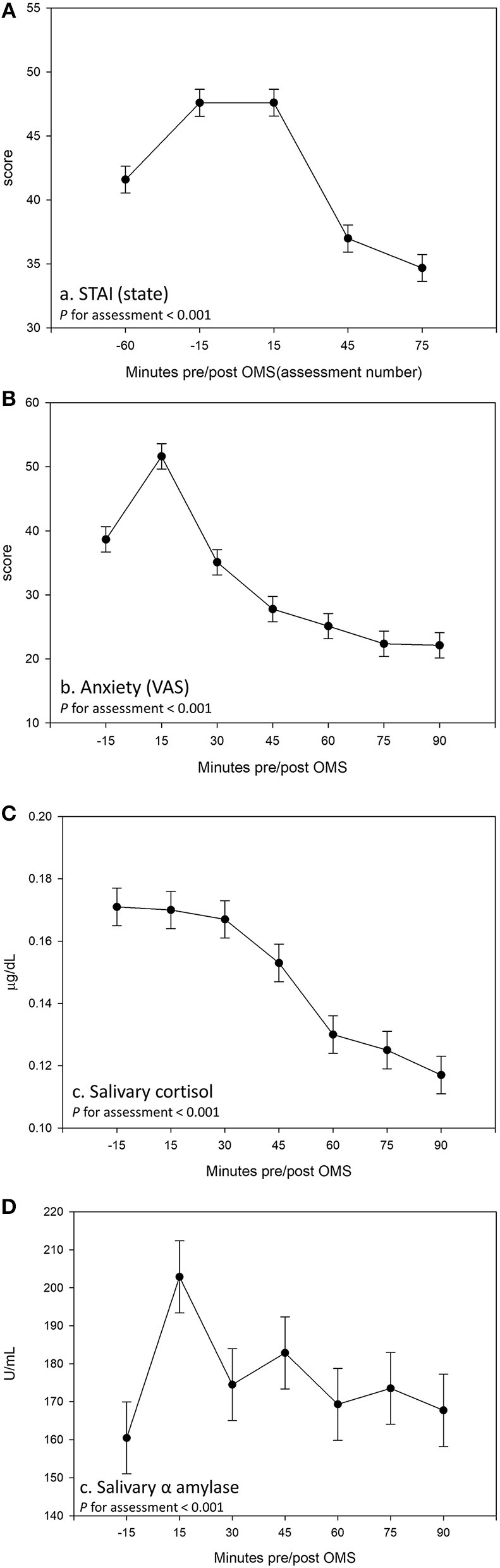
Effect of the Observed Multitasking Stressor (OMS) on **(A)** State–Trait Anxiety Inventory (STAI) (State), **(B)** anxiety measured using visual analog scale (VAS), **(C)** salivary cortisol, and **(D)** salivary α amylase. Data are pooled across treatment groups and visits. Estimated means ± standard error are shown derived from a linear mixed model including the terms treatment, visit, and assessment.

Analysis of the VAS and STAI-State revealed that all participants, irrespective of treatment, experienced an increase in subjective anxiety in response to the OMS protocol, which was coupled with an effect of assessment for salivary cortisol and α amylase, indicative of a physiological stress response (effect of assessment *p* < 0.001 for all outcomes, see Figure 6).

### Acute Response to the Stressor

**Figure 7:**
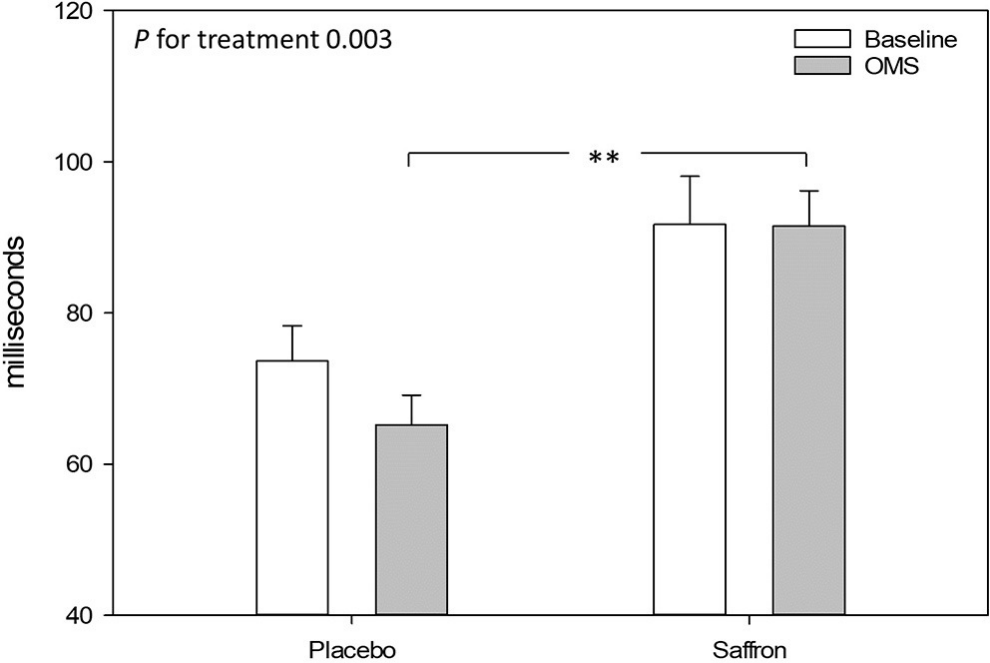
Effect of treatment on heart rate variability (root mean square of the successive differences) during the resting 5-min baseline and observed multitasking stressor (OMS). Estimated means ± standard error are shown. **p < 0.01.

Taking into account the baseline measure, a significant treatment effect was observed for the heart rate variability, RMSSD during OMS. Participants who received placebo had a decrease in RMSSD during OMS, whereas participants receiving saffron experienced no change in RMSSD [*F*_(1,44.1)_ = 9.92, *p* = 0.003]. See Figure 7.

**Figure 8:**
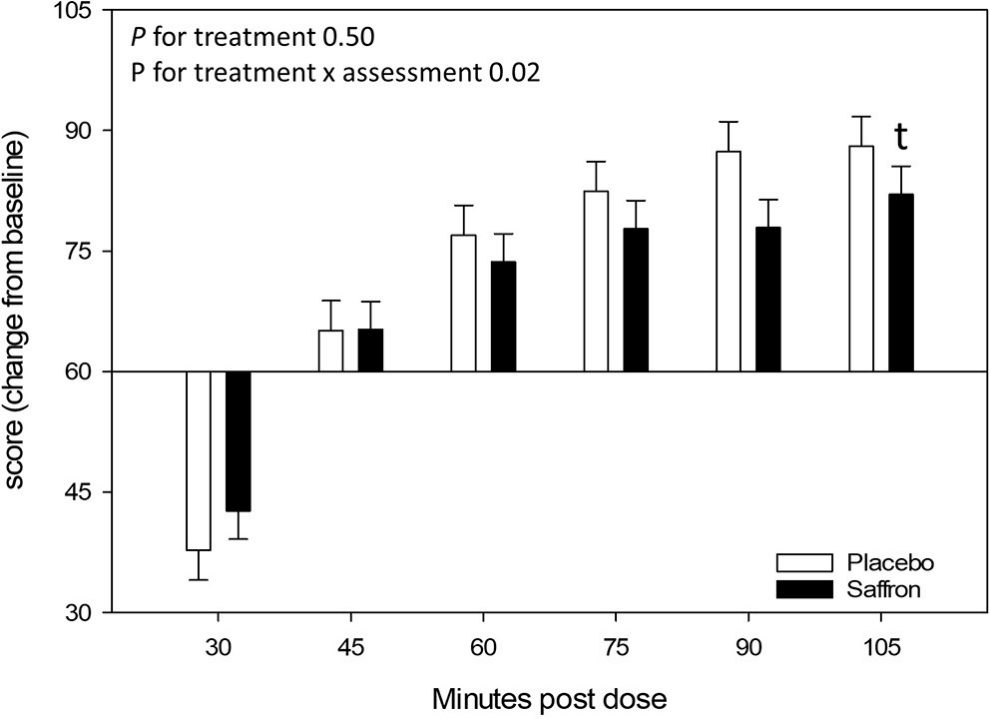
Effect of treatment on the Relaxed visual analog scale (VAS) at each assessment point. Estimated means ± standard error are shown. *t, p* < 0.10.

A significant Treatment × Assessment interaction was observed for the relaxed VAS [*F*_(5,955.05)_ = 2.75, *p* = 0.02]. Regardless of visit, pairwise comparisons revealed a trend for participants who received saffron to rate themselves as feeling less relaxed 105 min postdose (*p* = 0.07). See Figure 8.

With regards to salivary cortisol, the Friedman test within the saffron treatment group indicated that the change in Tmax salivary cortisol concentration from baseline was different across visits [Friedman's Q (3) = 8.72, *p* = 0.03]. However, *post*-hoc pairwise comparisons did not reveal any significant differences between visits (data not tabulated).

No other effects of Treatment nor Visit × Treatment interaction were observed. With regards safety, equal numbers of participants reported adverse events (20 in each group). None of these adverse events were serious, and none of them led to premature withdrawal or exclusion from the study. However, there was a greater number of incidents of gastrointestinal upset in the saffron group compared to the placebo group (5 vs. 0).

Tables of all outcome measures can be found in the Online Supplementary Material.

## Discussion

The current study explored the effects of a proprietary saffron extract (Safr'Inside™) on emotional well-being following 14, 28, and 56 days supplementation in healthy adults experiencing low mood as measured using the POMS. Effects of the extract on physiological and psychological response to a psychosocial stressor (OMS) were also measured following a single acute dose and postdose at the time points mentioned above. The results revealed several beneficial effects of saffron on subjective mood and quality of life measures, with the clearest finding reflecting a greater reduction in the depression scale of the POMS compared to placebo. This was accompanied by an improvement in social relationships on day 56. In addition, the stress-induced reduction in HRV (RMSSD) during the OMS observed in the placebo group was absent in those receiving saffron.

Turning first to the general mood measures that were collected predose on each of the study visits, compared to placebo, participants who received saffron experienced a greater reduction in depression scores. This finding reflects a growing body of evidence supporting the use of saffron in the treatment of depression in patient populations (6, 33). However, demonstration of the efficacy of saffron extract supplementation on depressive symptoms in a healthy population self-reporting low mood is completely new. Indeed, to our knowledge, only one other study has reported a positive effect of saffron on depressive symptoms in a healthy population (34). The comparable effects of saffron to other pharmacological treatments of depression has led to the conclusion that it may act on similar neurotransmitter systems, although the exact mechanisms are yet to be clarified (35). For example, Wang et al. (36) identified crocin 1 in an aqueous extract of saffron stigma, the administration of which was associated with antidepressant-like effects in mice who performed a forced swimming test (reduced immobility time). Crocin was also identified as an antagonist of the non-selective serotonin receptor agonist mCPP, which is known to induce excessive grooming in rats, and exacerbate symptoms in human patients with obsessive compulsive disorders (37). Other studies concluded that saffron may act by a combination of serotoninergic, noradrenergic, and dopaminergic system activation (38). In our study, we found that changes in urinary crocetin, probably due to changes in blood saffron metabolites, were strongly associated with variation in POMS depression T-score. This novel finding adds further evidence linking specific bioactive constituents of saffron with subjective mood in humans.

A positive effect of saffron on social relationships ratings was observed at day 56. This finding could be interpreted in the context of the concurrent improvement in depression scores observed in participants who received saffron. Previously, it has been shown that participants who exhibit greater depressive symptoms also report a higher number of negative social interactions and a lower sense of belonging in social interactions (39). It follows then that an improvement in depressive mood may manifest in enhanced social relationships. What is less clear is why the aforementioned change in mood would not be reflected in improvements in other quality of life measures. As this is the first time an effect of saffron on an individual's life beyond their mood state has been observed, further investigation is recommended.

Concerning the other measures, no evidence of an effect of saffron on subjective anxiety as assessed by the STAI questionnaire or anxiety scale of the HADS was found. An anxiolytic effect of saffron has previously been demonstrated in preclinical studies (37, 40) and in patients with mild to moderate depression (38, 41). Further, reduced tension (POMS) was reported by Kell et al. (34), suggesting a less consistent anxiolytic effect of saffron in healthy individuals self-reporting low mood. With regards to the current study, the context in which the predose mood and well-being questionnaires were completed by participants may also be important. Participants completed these questionnaires in full knowledge that they were going to complete the OMS, and this may have influenced their responses on these questionnaires. Subjective well-being has been shown to correlate with current mood (42) and also to be affected by experimental manipulation (43). It may be that anticipation of the OMS, evidenced by the pattern of response in the cortisol measure, masked an effect of treatment on anxiety measures or indeed any of the other subjective measures and is a limitation of the current design.

Although it seems that participants had anticipated the stressor, analysis of OMS data revealed that all participants, irrespective of treatment, found the protocol to be indeed stressful, evidenced by the observed increase in subjective anxiety immediately after completing the stressor. The significant effects of saffron in response to this psychosocial stressor were limited to attenuating a decrease in the HRV measure, RMSSD. This time-domain measure of beat-to-beat variability is thought to be primarily mediated by activity of the parasympathetic nervous system (PNS) (44) and is reduced during acute stress (45). Variation in RMSSD in response to OMS in the placebo group was in accordance with those observed following a moderate psychosocial stressor (45, 46). It is interesting that this measure of physiological response to stress was the only parameter to be modulated by saffron and perhaps suggests a specificity of the action of saffron on the PNS. Specifically, other HRV measures and GSR are predominantly modulated by sympathetic activity or antagonistic control of both the sympathetic nervous system and the PNS (44, 47, 48). Acetylcholine is the predominant neurotransmitter of the PNS, and it is possible that the observed effect of saffron, and specifically the constituent safranal, is underpinned by increased cholinergic activity (49). Anxiolytic effects in response to an acute lab-based stressor have also been demonstrated following monoterpenoid plant extracts such as those derived from sage and lemon balm (50, 51). Interestingly, an acute 200 μl dose of *Lavandula angustifolia* (lavender) oil—a source of the monoterpene linalool—modulated RMSSD during viewing of an anxiety-inducing film clip, but only in females (52). However, reduced GSR, HR, and increased HRV (pNN50, which correlates highly with RMSSD) were reported in all participants following a neutral film clip. The authors suggest that lavender may modulate serotonin pathways that interact with cholinergic systems in a similar fashion as selective serotonin reuptake inhibitors (SSRIs) such as fluoxetine and citalopram, given evidence that HRV increases following treatment with this class of drugs in patients with panic disorder (53). The similar pattern of effects of saffron compared to SSRIs in the treatment of depression in clinical trials (9) certainly adds weight to this suggestion with regard the current findings.

Safranal may also modulate HRV through its action on GABAergic systems (54). In one study, peripherally administered safranal abolished the seizure effects of pentylenetetrazol that the authors suggested could be mediated via the GABA_A_-benzodiazepine receptor complex (55). These authors also noted that related monoterpenes such as linalool and terpineol potentiate GABA and competitively inhibit glutamate receptors, further strengthening this argument. Interestingly, acute administration of both normal and GABA-fortified oolong tea was found to increase RR intervals and decrease subjective ratings of stress in healthy participants, with a greater effect seen in the GABA-fortified tea condition (56). As HRV is a measure that reflects autonomic health and an indicator of psychological resilience (44), the observed effect of saffron on RMSSD reported here may have far-reaching health benefits. It would be interesting to investigate whether this effect was observed over a longer period (e.g., 24-h monitoring), suggesting modulation of overall parasympathetic tone, or if the effect is only observed during acute stress.

Of course, the study is not without its limitations. It is recognized that laboratory-based stressors have inherent limitations in eliciting robust psychobiological stress responses that mimic those experienced in real-world situations—especially with repeated measurement—which have been discussed elsewhere (57). The nature of the study sample should also be considered. Identifying potential participants with subclinical mood disturbance resulted in a comparatively small number of individuals with a very specific response pattern to the screening questionnaires being enrolled to the trial. Although it was the aim of the study to investigate the effects of saffron in otherwise healthy participants with subclinical mood disorders, it does of course limit the relevance of the findings beyond this population, and the effects in wider general or clinical populations are unknown. Furthermore, the inclusion and exclusion criteria themselves were not based on formal diagnostic criteria, and the link between the selected criteria and risk of developing depression is also unknown. Lastly, while we attempted to limit the risk of bias by excluding certain conditions or lifestyle behaviors, it must be acknowledged that it is also important to examine the effectiveness of saffron and whether its effects are increased or diminished when coupled with other mood-associated variables.

In conclusion, saffron extract appears to reduce depressive mood in healthy individuals experiencing subclinical mood disturbance and adds to the growing literature showing consistent benefits of saffron on depression outcomes across both clinical and non-clinical populations. Importantly, the beneficial effect of saffron on heart rate variability in response to a psychosocial stressor—shown for the very first time in the present study—suggests that this natural extract may be particularly relevant for increasing resilience against the development of stress-related psychiatric disorders. Further research is needed to identify the exact mechanisms underpinning these effects in humans.

## Data Availability Statement

The raw data supporting the conclusions of this article will be made available by the authors, without undue reservation.

## Ethics Statement

The studies involving human participants were reviewed and approved by Northumbria University Department of Psychology Ethics Committee. The patients/participants provided their written informed consent to participate in this study.

## Author Contributions

PJ, DK, JF, JK, CP, SD, DG, BM, LP, FJ, CV, KB, HA, JB, JC, DV, and LC made a substantial contribution to the concept and design, acquisition of data or analysis, and interpretation of data. PJ compiled the first draft of the article. All authors were involved in reviewing the manuscript for publication and approved the version to be published.

## Conflict of Interest

CP, SD, DG, BM, and LP are employees of Activ'Inside. The remaining authors declare that the research was conducted in the absence of any commercial or financial relationships that could be construed as a potential conflict of interest.

## References

[1] World Health Organisation Depression and Other Common Mental Disorders. Geneva: Global Health Estimates (2017).

[2] Cuijpers P de Graaf R van Dorsselaer S. Minor depression: risk profiles, functional disability, health care use and risk of developing major depression. J Affect Disord. (2004) 79:71–9. 10.1016/S0165-0327(02)00348-8 15023482

[3] Fournier JC DeRubeis RJ Hollon SD Dimidjian S Amsterdam JD Shelton RC. Antidepressant drug effects and depression severity: a patient-level meta-analysis. JAMA. (2010) 303:47–53. 10.1001/jama.2009.1943 20051569 PMC3712503

[4] Braun C Adams A Rink L Bschor T Kuhr K Baethge C. In search of a dose–response relationship in SSRIs—a systematic review, meta-analysis, and network meta-analysis. Acta Psychiatr Scand. (2020) 142:430–42 10.1111/acps.13235 32970827

[5] Qureshi NA Al B. Mood disorders and complementary and alternative medicine: a literature review. Neuropsychiatr Dis Treat. (2013) 9:639–58. 10.2147/NDT.S43419 23700366 PMC3660126

[6] Lopresti AL Drummond PD. Saffron (*Crocus sativus*) for depression: a systematic review of clinical studies and examination of underlying antidepressant mechanisms of action. Human Psychopharmacol. (2014) 29:517–27. 10.1002/hup.2434 25384672

[7] Rios JL Recio MC Giner RM Manez S An update review of saffron and its active constituents. Phytother Res. (1996) 10:189–93. 10.1002/(SICI)1099-1573(199605)10:3<189::AID-PTR754>3.0.CO;2-C

[8] Akhondzadeh S Tahmacebi-Pour N Noorbala AA Amini H Fallah-Pour H Jamshidi AH. *Crocus sativus L*. in the treatment of mild to moderate depression: a double-blind, randomized and placebo-controlled trial. Phytother Res. (2005) 19:148–51. 10.1002/ptr.1647 15852492

[9] Hausenblas HA Saha D Dubyak PJ Anton SD. Saffron (*Crocus sativus L.*) and major depressive disorder: a meta-analysis of randomized clinical trials. J Integr Med. (2013) 11:377–83. 10.3736/jintegrmed2013056 24299602 PMC4643654

[10] Leonardo ED Hen R Anxiety as a developmental disorder. Neuropsychopharmacology. (2008) 33:134–40. 10.1038/sj.npp.1301569 17851538

[11] Charney DS. Psychobiological mechanisms of resilience and vulnerability: implications for successful adaptation to extreme stress. Am J Psychiatry. (2004) 161:195–216. 10.1176/appi.ajp.161.2.195 14754765

[12] Varghese FP Brown ES. The hypothalamic-pituitary-adrenal axis in major depressive disorder: a brief primer for primary care physicians. Prim Care Companion J Clin Psychiatry. (2001) 3:151–5. 10.4088/PCC.v03n0401 15014598 PMC181180

[13] Ghadrdoost B Vafaei AA Rashidy-Pour A Hajisoltani R Bandegi AR Motamedi F. Protective effects of saffron extract and its active constituent crocin against oxidative stress and spatial learning and memory deficits induced by chronic stress in rats. Eur J Pharmacol. (2011) 667:222–9. 10.1016/j.ejphar.2011.05.012 21616066

[14] Hooshmandi Z Rohani AH Eidi A Fatahi Z Golmanesh L Sahraei H. Reduction of metabolic and behavioral signs of acute stress in male Wistar rats by saffron water extract and its constituent safranal. Pharm Biol. (2011) 49:947–54. 10.3109/13880209.2011.558103 21592014

[15] Fukui H Toyoshima K Komaki R. Psychological and neuroendocrinological effects of odor of saffron (*Crocus sativus*). Phytomedicine. (2011) 18:726–30. 10.1016/j.phymed.2010.11.013 21242071

[16] Cuijpers P Smit F. Subthreshold depression as a risk indicator for major depressive disorder: a systematic review of prospective studies. Acta Psychiatr Scand. (2004) 109:325–31. 10.1111/j.1600-0447.2004.00301.x 15049768

[17] Meyer TJ Miller ML Metzger RL Borkovec TD. Development and validation of the penn state worry questionnaire. Behav Res Ther. (1990) 28:487–95. 10.1016/0005-7967(90)90135-6 2076086

[18] Cohen S Kamarck T Mermelstein R. A global measure of perceived stRESS. J Health Soc Behav. (1983) 24:385–96. 10.2307/2136404 6668417

[19] Spitzer RL Kroenke K Williams JB Lowe B. A brief measure for assessing generalized anxiety disorder: the GAD-7. Arch Intern Med. (2006) 166:1092–7. 10.1001/archinte.166.10.1092 16717171

[20] Kroenke K Spitzer RL Williams JB. The PHQ-9: validity of a brief depression severity measure. J Gen Intern Med. (2001) 16:606–13. 10.1046/j.1525-1497.2001.016009606.x 11556941 PMC1495268

[21] Speilberger CD Gorsuch RL Lushene RE. The State Trait Anxiety Inventory Manual. Palo Alto, CA: Consulting Psychologists Press (1969).

[22] Heuchert JP McNair DM POMS 2 Manual: Profile of Mood States. Toronto, ON: Multi-Health Systems Inc (2012) 10.1037/t05057-000

[23] Kennedy DO Bonnländer B Lang SC Pischel I Forster J Khan J. Acute and chronic effects of green oat (Avena sativa) extract on cognitive function and mood during a laboratory stressor in healthy adults: a randomised, double-blind, placebo-controlled study in healthy humans. Nutrients. (2020) 12:1598. 10.3390/nu12061598 32485993 PMC7352613

[24] Carver CS Scheier MF Weintraub JK. Assessing coping strategies: a theoretically based approach. J Pers Soc Psychol. (1989) 56:267–83. 10.1037/0022-3514.56.2.267 2926629

[25] Zigmond AS Snaith RP. The hospital anxiety and depression scale. Acta Psychiatr Scand. (1983) 67:361–70. 10.1111/j.1600-0447.1983.tb09716.x 6880820

[26] WHOQOL group Development of the World Health Organization WHOQOL-BREF quality of life assessment. Psychol Med. (1998) 28:551–8. 10.1017/S0033291798006667 9626712

[27] Zhang Y Fei F Zhen L Zhu X Wang J Li S. Sensitive analysis and simultaneous assessment of pharmacokinetic properties of crocin and crocetin after oral administration in rats. J Chromatogr B Analyt Technol Biomed Life Sci. (2017) 1044–1045:1–7. 10.1016/j.jchromb.2016.12.003 28056427

[28] Nazaraghaei F Bhat KK Physiological impacts of Ajapajapa Yogic Meditation on HRV index, RMSSD, PNN50, Heart Rate and GSR following three-month training course in comparison to F.G. Meditation. J Adv Med Sci Appl Technol. (2020) 5:1–9. 10.30476/JAMSAT.2020.46603

[29] Nazaraghaie F Torkamani F Kiani B Nami M Research paper: quantitative electroencephalogram-informed geometric meditation: a pilot validation study. J Adv Med Sci Appl Technol. (2017) 3:163–8. 10.32598/jamsat.3.3.163

[30] Zhang Y Huo M Zhou J Xie S. PKSolver: an add-in program for pharmacokinetic and pharmacodynamic data analysis in Microsoft Excel. Comput Methods Programs Biomed. (2010) 99:306–14. 10.1016/j.cmpb.2010.01.007 20176408

[31] Brouns F Bjorck I Frayn KN Gibbs AL Lang V Slama G. Glycaemic index methodology. Nutr Res Rev. (2005) 18:145–71. 10.1079/NRR2005100 19079901

[32] Succop PA Clark S Chen M Galke W. Imputation of data values that are less than a detection limit. J Occup Environ Hyg. (2004) 1:436–41. 10.1080/15459620490462797 15238313

[33] Marx W Lane M Rocks T Ruusunen A Loughman A Lopresti A. Effect of saffron supplementation on symptoms of depression and anxiety: a systematic review and meta-analysis. Nutr Rev. (2019) 77:557–71. 10.1093/nutrit/nuz023 31135916

[34] Kell G Rao A Beccaria G Clayton P Inarejos-Garcia AM Prodanov M affron(R) a novel saffron extract (*Crocus sativus L*) improves mood in healthy adults over 4 weeks in a double-blind, parallel, randomized, placebo-controlled clinical trial. Complement Ther Med. (2017) 33:58–64. 10.1016/j.ctim.2017.06.001 28735826

[35] Hausenblas HA Heekin K Mutchie HL Anton S. A systematic review of randomized controlled trials examining the effectiveness of saffron (*Crocus sativus L.*) on psychological and behavioral outcomes. J Integr Med. (2015) 13:231–40. 10.1016/S2095-4964(15)60176-5 26165367 PMC5747362

[36] Wang Y Han T Zhu Y Zheng CJ Ming QL Rahman K. Antidepressant properties of bioactive fractions from the extract of *Crocus sativus L*. J Nat Med. (2010) 64:24–30. 10.1007/s11418-009-0360-6 19787421

[37] Georgiadou G Tarantilis PA Pitsikas N Effects of the active constituents of *Crocus Sativus L.*, crocins, in an animal model of obsessive-compulsive disorder. Neurosci Lett. (2012) 528:27–30. 10.1016/j.neulet.2012.08.081 22985509

[38] Mazidi M Shemshian M Mousavi SH Norouzy A Kermani T Moghiman T. A double-blind, randomized and placebo-controlled trial of Saffron (*Crocus sativus L.*) in the treatment of anxiety and depression. J Complement Integrat Med. (2016) 13:195–9. 10.1515/jcim-2015-0043 27101556

[39] Steger MF Kashdan TB. Depression and everyday social activity, belonging, and well-being. J Couns Psychol. (2009) 56:289–300. 10.1037/a0015416 20428460 PMC2860146

[40] Pitsikas N Boultadakis A Georgiadou G Tarantilis PA Sakellaridis N Effects of the active constituents of *Crocus sativus L.*, crocins, in an animal model of anxiety. Phytomedicine. (2008) 15:1135–9. 10.1016/j.phymed.2008.06.005 18693098

[41] Talaei A Hassanpour Moghadam M Sajadi Tabassi SA Mohajeri SA. Crocin, the main active saffron constituent, as an adjunctive treatment in major depressive disorder: a randomized, double-blind, placebo-controlled, pilot clinical trial. J Affect Disord. (2015) 174:51–6. 10.1016/j.jad.2014.11.035 25484177

[42] Yardley JK Rice RW The relationship between mood and subjective well-being. Soc Indic Res. (1991) 24:101–11. 10.1007/BF00292653

[43] Yap SCY Wortman J Anusic I Baker SG Scherer LD Donnellan MB. The effect of mood on judgments of subjective well-being: nine tests of the judgment model. J Pers Soc Psychol. (2017) 113:939–61. 10.1037/pspp0000115 27936835 PMC5899828

[44] Shaffer F McCraty R Zerr CL. A healthy heart is not a metronome: an integrative review of the heart's anatomy and heart rate variability. Front Psychol. (2014) 5:1040. 10.3389/fpsyg.2014.01040 25324790 PMC4179748

[45] Castaldo R Melillo P Bracale U Caserta M Triassi M Pecchia L Acute mental stress assessment via short term HRV analysis in healthy adults: a systematic review with meta-analysis. Biomed Signal Process Control. (2015) 18:370–7. 10.1016/j.bspc.2015.02.012

[46] Brugnera A Zarbo C Tarvainen MP Marchettini P Adorni R Compare A. Heart rate variability during acute psychosocial stress: a randomized cross-over trial of verbal and non-verbal laboratory stressors. Int J Psychophysiol. (2018) 127:17–25. 10.1016/j.ijpsycho.2018.02.016 29501671

[47] Sugenoya J Iwase S Mano T Ogawa T. Identification of sudomotor activity in cutaneous sympathetic nerves using sweat expulsion as the effector response. Eur J Appl Physiol Occup Physiol. (1990) 61:302–8. 10.1007/BF00357617 2282916

[48] Wang C-A Baird T Huang J Coutinho JD Brien DC Munoz DP. Arousal effects on pupil size, heart rate, and skin conductance in an emotional face task. Front Neurol. (2018) 9:1029. 10.3389/fneur.2018.01029 30559707 PMC6287044

[49] Geromichalos GD Lamari FN Papandreou MA Trafalis DT Margarity M Papageorgiou A. Saffron as a source of novel acetylcholinesterase inhibitors: molecular docking and *in vitro* enzymatic studies. (2012) 60:6131–8. 10.1021/jf300589c 22655699

[50] Kennedy DO Little W Scholey AB. Attenuation of laboratory-induced stress in humans after acute administration of Melissa officinalis (lemon balm). Psychosom Med. (2004) 66:607–13. 10.1097/01.psy.0000132877.72833.71 15272110

[51] Kennedy DO Pace S Haskell C Okello EJ Milne A Scholey AB. Effects of cholinesterase inhibiting sage (Salvia officinalis) on mood, anxiety and performance on a psychological stressor battery. Neuropsychopharmacology. (2006) 31:845–52. 10.1038/sj.npp.1300907 16205785

[52] Bradley BF Brown SL Chu S Lea RW. Effects of orally administered lavender essential oil on responses to anxiety-provoking film clips. Human Psychopharmacol. (2009) 24:319–30. 10.1002/hup.1016 19382124

[53] Gorman JM Sloan RP. Heart rate variability in depressive and anxiety disorders. Am Heart J. (2000) 140 (4, Suppl):S77–83. 10.1067/mhj.2000.109981 11011352

[54] Hosseinzadeh H Talebzadeh F. Anticonvulsant evaluation of safranal and crocin from *Crocus sativus* in mice. Fitoterapia. (2005) 76:722–4. 10.1016/j.fitote.2005.07.008 16253437

[55] Hosseinzadeh H Sadeghnia HR. Protective effect of safranal on pentylenetetrazol-induced seizures in the rat: involvement of GABAergic and opioids systems. Phytomedicine. (2007) 14:256–62. 10.1016/j.phymed.2006.03.007 16707256

[56] Hinton T Jelinek HF Viengkhou V Johnston GA Matthews S. Effect of GABA-Fortified oolong tea on reducing stress in a university student cohort. Front Nutrition. (2019) 6:27. 10.3389/fnut.2019.00027 30972340 PMC6443991

[57] Wetherell MA Craw O Smith K Smith MA. Psychobiological responses to critically evaluated multitasking. Neurobiol Stress. (2017) 7:68–73. 10.1016/j.ynstr.2017.05.002 28540348 PMC5432679

