# Supplementary material for: Effects of Saffron Extract Supplementation on Mood, Well-Being, and Response to a Psychosocial Stressor in Healthy Adults: A Randomized, Double-Blind, Parallel Group, Clinical Trial
Source: Front Nutr. 2021 Feb 1;7:606124. doi: 10.3389/fnut.2020.606124 (PMC7882499; doi:10.3389/fnut.2020.606124)
Supplement: Supplementary file 1 [file Table_1.DOCX]

**Supplemental file 1. Full list of inclusion/exclusion criteria**

**INCLUSION CRITERIA**

- Healthy male or female adults (at least 35% of each gender)
- Aged between 18 and 60 years inclusive
- With non-pathological feelings of anxiety and/ or stress in daily life:

- Subjects self-reporting low mood;

- Total score ≥ 40 at the Profile of Mood State (POMS 2);

- Score < 16 at the Generalized Anxiety Disorder 7-item (GAD-7) questionnaire, so that volunteers with moderate to severe anxiety could not be included

- Score ≤ 10 at the Patient Health Questionnaire 9-item (PHQ-9), so that volunteers with moderate to severe depression could not be included

- Not meeting the diagnosis criteria for any mental disorder (depression, generalised anxiety disorder, obsessive compulsive disorder, panic disorder, simple phobia and social phobia, schizophrenia, etc.)

- Body Mass Index (BMI) in the normal range: 18.5 ≥ BMI ≤ 30 kg/ m2
- For non-menopausal women: using effective contraception/pregnancy is not physiologically possible. If pharmacological contraception in progress: it must have been started at least 3 cycles beforehand at stable doses and must remain unchanged for the entire duration of the study
- For post-menopausal women: with or without HRT. If hormone replacement therapy in progress: it must be stable still at least 3 months and should not be changed during the study. Women within the menopausal transition (peri-menopause) are not eligible (i.e. irregular menstruations with clinical symptoms of menopause such as hot flushes, mood and/ or sleep disorders **and/or** amenorrhea for more than one month but less than 12 months)
- Subject showing no difficulty for salivary sampling (a first try will be performed during the inclusion visit in the presence of the investigator)
- Subjects have a bank account (required for payment)
- Subjects capable of and willing to comply with the protocol and to give their written informed consent

**EXCLUSION CRITERIA**

Subjects complying with at least one of the following criteria will not be eligible:

- Diagnosis of psychological pathology (depression, generalised anxiety disorder, obsessive compulsive disorder, panic disorder, simple phobia, social phobia, schizophrenia, etc.) within the previous 3 years
- Diagnosis of cognitive pathology (Alzheimer’s disease, Parkinson’s disease, dementia)
- Anxiolytic, antidepressant or any other treatment likely to affect some of the study parameters, whatever the reason of its prescription, within the previous 3 months
- Event (personal or professional) likely to have impacted the subject’s emotional and/ or psychological state within the last 8 weeks (for example but not restricted to: change of professional function/situation, death of a family member, divorce, surgery, accident, etc.)
- Event (personal or professional) likely to affect the subject’s emotional, psychological or hormonal state planned during the next 8 weeks, including vaccination, important medical exam etc.
- Hormonal state likely to induce an unstable/fluctuating emotional state during the study, such as but not restricted to: post-partum period (< 6 months after delivery) and menopausal transition (irregular menstrual cycle and mood disorders and/or hot flushes)
- Have high blood pressure (systolic over 159 mm Hg or diastolic over 99 mm Hg)
- Subjects diagnosed with at least one of the following will not be eligible:
  - diabetes (type I or type II),
  - and/ or cardiovascular disease or history of cardiovascular disease (such as atherosclerosis, heart disease),
  - and/ or present or recurrent infectious diseases (including cystitis, gingivitis, conjunctivitis, respiratory infections);
  - chronic inflammatory pathology (rheumatoid arthritis, arthrosis, etc.) or allergic disease (asthma, allergic rhinitis, atopic dermatitis);
  - any other pathology which, according to investigator’s judgment, is likely to affect the study parameters.
- Diagnosed gastrointestinal disease or disorder likely to alter intestinal absorption, such as but not restricted to: Crohn’s disease, celiac disease, ulcerative colitis, irritable bowel syndrome, symptomatic diverticulosis, lactose intolerance, history of intestinal resection etc.
- Usual corticoid treatment/ steroidal anti-inflammatory treatment (ex: Betamethasone, Cortivazol, Dexamethasone, Methylprednisolone, Prednisolone, Prednisone, Tétracosactide, Triamcinolone, etc.) including local treatments (ex: Locoïd lotion, Locatop, Locapred, Tridesonit, etc.). Subjects will not be eligible if they have consumed such treatments within the last 2 weeks before V1 and/or if they are likely to consume such treatments during the study
- Oral antibiotic treatment within the last month before entry into the study. In case of oral antibiotic started after inclusion, this treatment will be reported as deviation but the subject will not be excluded
- Unbalanced thyroid disease. However, subjects with controlled thyroid diseases (medication unchanged within the last 3 months) can be included
- Suffering from a severe chronic pathology which, according to Investigator’s judgment, is likely to affect at least one of the study parameters (such as but not restricted to: severe chronic pain, cancer or history of cancer unless in remission for more than 5 years, HIV, hepatitis, renal disease, cardiac disease)
- High physical activity practice: more than 10 hours per week of moderate to vigorous physical activity, or physical activity practice significantly modified since less than 2 months or likely to be modified within the next 8 weeks. Examples of moderate physical activity are: transporting light loads, bicycling at a normal pace, double tennis etc. Walking is not considered as physical activity of moderate intensity
- Subjects working in shift schedules (ex: nurse, baker, etc.)
- Subjects whose working conditions are variable and likely to be extreme (ex: work in a cold room)
- Tobacco consumption during a normal 24-hour period (smoking restricted to social occasions is allowed) or change in smoking habits within the previous 2 months or planned within the next 8 weeks
- Subjects consuming any food supplement (including vitamins, minerals and botanicals and/or other substances) and refusing to stop for at least 2 weeks before V1 (inclusion visit) and until the end of his/ her study participation
- Subjects having a recent history of (within 3 months of screening visit) or strong potential for alcohol abuse. Alcohol abuse is defined as more than 60g (men) / 40g (women) pure alcohol per day (7 / 5.5 units)
- Excessive caffeine use (> 500 mg/d)
- Consumption of recreational drugs
- Travel (for any reason) in a foreign country resulting in a jetlag within 15 days before V1
- Subjects planning a holiday of ≥7 days between V1 and V3 or ≥ 14 days between V3 and V4 (subject must have a 7 day ‘washout’ following holidays ≥7 days).
- Change in dietary habits since less than 4 weeks before V1 or planned within the next 10 weeks of the trial period
- Eating disorders: anorexia and bulimia or unstable dietary pattern;
- Pregnancy in progress (or suspected/ risky)
- Any food allergy documented or suspected to one of the components of the study products
- Subject presenting a psychological or linguistic inability to sign the informed consent;
- Subject under legal protection (guardianship, wardship) or deprived from his rights following administrative or judicial decision
- Subject impossible to contact in case of emergency
- Subject has a visual impairment that cannot be corrected with glass or lenses
