# Supplementary material for: Effects of Saffron Extract Supplementation on Mood, Well-Being, and Response to a Psychosocial Stressor in Healthy Adults: A Randomized, Double-Blind, Parallel Group, Clinical Trial
Source: Front Nutr. 2021 Feb 1;7:606124. doi: 10.3389/fnut.2020.606124 (PMC7882499; doi:10.3389/fnut.2020.606124)
Supplement: Supplementary file 3 [file Table_3.DOCX]

Table 1. Performance on the serial subtractions and tracking tasks completed during the OMS

Data are estimated means and standard error derived from the linear mixed model analysis, with the final two columns showing effects (F) and associated probabilities (p). Tr=main effect of treatment; Tr x V=treatment x visit interaction effect; Tr x Ta=treatment x task interaction effect; Tr x V x Ta= treatment x visit x task interaction effect. The first N column reflects the number of participants included in the final analysis, N for Visit indicates the number of available values at each of the study assessments.

Table 2. Physiological response to the OMS.

The baseline data are raw means (plus SEM) collected during a pre-OMS 5-minute rest period. Data presented from the subtractions tasks (3s, 7s, 17s) are estimated means (plus SEM) of change from baseline values derived from the linear mixed model analysis, with the final two columns showing effects (F) and associated probabilities (p). Tr=main effect of treatment; Tr x V=treatment x visit interaction effect; Tr x Ta=treatment x task interaction effect; Tr x V x Ta= treatment x visit x task interaction effect. The first N column reflects the number of participants included in the final analysis, N for Baseline and task indicates the number of available values at each of the study assessments.

Table 3. Psychological response to the OMS (visual analogue scales).

The baseline data are raw means (plus SEM) collected during the pre-OMS assessment. Data presented from 30 to 105 minutes post dose are estimated means (plus SEM) of change from baseline values derived from the linear mixed model analysis, with the final two columns showing effects (F) and associated probabilities (p). Tr=main effect of treatment; Tr x V=treatment x visit interaction effect; Tr x A=treatment x assessment interaction effect; Tr x V x A= treatment x visit x assessment interaction effect. The first N column reflects the number of participants included in the final analysis, N for Baseline and minutes post dose indicates the number of available values at each of the study assessments.

Table 4. Psychological response to the OMS (STAI-State).

The baseline data are raw means (plus SEM) collected during the pre-OMS assessment. Data presented from 30 to 90 minutes post dose are estimated means (plus SEM) of change from baseline values derived from the linear mixed model analysis, with the final two columns showing effects (F) and associated probabilities (p). Tr=main effect of treatment; Tr x V=treatment x visit interaction effect; Tr x A=treatment x assessment interaction effect; Tr x V x A= treatment x visit x assessment interaction effect. The first N column reflects the number of participants included in the final analysis, N for Baseline and minutes post dose indicates the number of available values at each of the study assessments.

Table 5. Chronic mood assessment assessed pre-dose at each testing visit.

The baseline data are raw means (plus SEM) collected pre-dose at Visit 1. Data presented from visits 2 to 4 are estimated means (plus SEM) of change from baseline values derived from the linear mixed model analysis, with the final two columns showing effects (F) and associated probabilities (p). Tr=main effect of treatment; Tr x V=treatment. The first N column reflects the number of participants included in the final analysis, N for Baseline and visit indicates the number of available values at each of the study assessments.

Table 6. Trait anxiety (STAI-trait) at visit 4.

The baseline data are raw means (plus SEM) collected at the screening visit (V0). Data presented from visit 4 are estimated means (plus SEM) of STAI-trait score. The first N column reflects the number of participants included in the final analysis, N for Baseline and Visit 4 indicates the number of available values at each of the study assessments. . P-value derived from Analysis of Covariance at Visit 4 using Visit 0 score as a covariate.

Table 7. Salivary α-amylase and cortisol area under the curve (AUC) calculations.

Data are estimated means (plus SEM) derived from the linear mixed model analysis, with the final two columns showing effects (F) and associated probabilities (p). Tr=main effect of treatment; Tr x V=treatment. The first N column reflects the number of participants included in the final analysis, N for Baseline and visit indicates the number of available values at each of the study assessments.

Table 8. Time (in minutes) of maximum concentration of salivary α-amylase and cortisol.

Data are median values with the final two columns showing columns showing Chi2 effects (χ2) and associated probabilities (p) comparing visit within each treatment group. The first N column reflects the number of participants included in the final analysis. LOD, level of detection.
